# Overexpression of Eag1 potassium channels in clinical tumours

**DOI:** 10.1186/1476-4598-5-41

**Published:** 2006-10-05

**Authors:** Bernhard Hemmerlein, Rüdiger M Weseloh, Fernanda Mello de Queiroz, Hendrik Knötgen, Araceli Sánchez, María E Rubio, Sabine Martin, Tessa Schliephacke, Marc Jenke, Heinz-Joachim-Radzun, Walter Stühmer, Luis A Pardo

**Affiliations:** 1 Department of Pathology, Georg-August University, Robert-Koch-Str. 40, 37075 Göttingen, Germany; 2 Max-Planck Institute of Experimental Medicine, Hermann-Rein-Str. 3, 37075 Göttingen, Germany; 3 Divisão de Farmacologia, Coordenação de Pesquisa, Instituto Nacional do Câncer, Rua André Cavalcanti 37/3° andar, Rio de Janeiro, Brasil; 4 iOnGen AG, Stiegbreite 13, 37077 Göttingen, Germany; 5 DFG Research Center for the Molecular Physiology of the Brain (CMPB), Göttingen, Germany

## Abstract

**Background:**

Certain types of potassium channels (known as Eag1, KCNH1, Kv10.1) are associated with the production of tumours in patients and in animals. We have now studied the expression pattern of the Eag1 channel in a large range of normal and tumour tissues from different collections utilising molecular biological and immunohistochemical techniques.

**Results:**

The use of reverse transcription real-time PCR and specifically generated monoclonal anti-Eag1 antibodies showed that expression of the channel is normally limited to specific areas of the brain and to restricted cell populations throughout the body. Tumour samples, however, showed a significant overexpression of the channel with high frequency (up to 80% depending on the tissue source) regardless of the detection method (staining with either one of the antibodies, or detection of Eag1 RNA).

**Conclusion:**

Inhibition of Eag1 expression in tumour cell lines reduced cell proliferation. Eag1 may therefore represent a promising target for the tailored treatment of human tumours. Furthermore, as normal cells expressing Eag1 are either protected by the blood-brain barrier or represent the terminal stage of normal differentiation, Eag1 based therapies could produce only minor side effects.

## Background

Ion channels play key roles in cellular functions other than electrical signal transmission [1]. In recent years, the importance of voltage-gated potassium channels in tumour biology has aroused interest with the identification of ion channels as potential novel targets for tumour therapy [2-5]. The first identified voltage-gated potassium channel implicated in oncogenesis and tumour progression was Eag1 [6].

Eag1 was first described as a cell-cycle regulated channel [7-9] relevant in the process of myoblast fusion [10,11], although the RNA is detected only in brain and placenta by Northern blot on human specimens [11], indicating that the channel is not expressed in differentiated peripheral tissues. In recent years, we have explored the role of Eag1 in the control of cell proliferation and found that it shows both transforming properties *in vitro *(i.e. it confers loss of contact inhibition and increased growth rate) and increases the speed of growth and the invasiveness of tumours implanted into SCID mice *in vivo *[6]. To date, Eag1 is the only potassium channel that has been shown to affect tumour progression in animal models. Eag1 protein expression has been detected in several cell lines derived from human malignant tumours, such as neuroblastoma [6,12], melanoma [13], and breast [6,14], and cervical carcinoma [6]. In these cell lines, Eag1 enhances the proliferation of the cells, and is required for the maintenance of growth. Moreover, specific inhibition of Eag1 expression by antisense technology [6], siRNA [15] or by non-specific blockers [14,16,17] leads to a reduction of tumour cell proliferation *in vitro*. Recently, functional expression of Eag1 has been described in clinical samples of cervical carcinoma, while the channel was absent in control samples devoid of pathological findings [18], and aberrant expression of the channel has also been detected in sarcomas [17], while the surrounding tissues were devoid of Eag1 expression.

For several reasons Eagl represents an interesting target for tumour therapy. This membrane protein is accessible from the extracellular side and is predominantly present in tumour cells. For any potential clinical application it is an essential pre-requisite that samples from human tumours (and not only cell lines) overexpress the target Eag1. For this reason, we performed immunohistochemical and real-time PCR experiments to determine the expression patterns of Eag1 within normal and neoplastic tissues in detail. We found very low expression levels in normal human tissues and an unusually high prevalence of Eag1 overexpression in various types of human malignant tumours.

## Results

### Eag1.62.mAb selectively detects Eag1 expression

Eag1.62.mAb was selected for immunohistochemistry based on its ability to bind Eag1 fusion protein and not Eag2 as determined by ELISA and Biacore analysis. The specificity of this antibody was then further tested in a number of other ways as outlined below.

**Figure 1:**
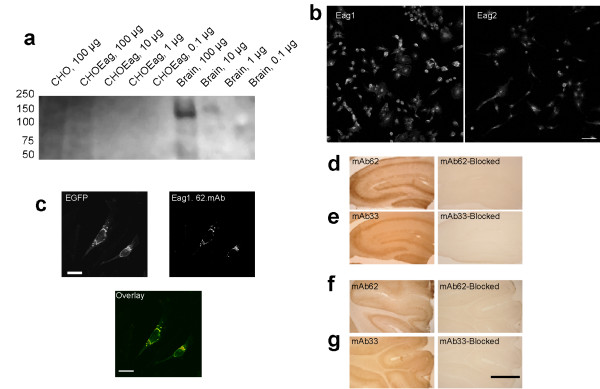
**Characterization of the Eag1 antibodies**. **a**. Western blot analysis of Eag1-expressing membrane preparations with anti-Eag1.62.mAb. A single protein is detected only when using large amounts of brain extract protein. **b**. Eag1.62.mAb stained CHO cells transfected with Eag1 (left) while cells transfected with Eag2 (right) show only faint background, indicating that Eag1.62.mAb does not recognise Eag2. **c**. CHO cells transiently expressing a chimera between EGFP and hEag1. Chimeras were stained with Eag1.62.mAb. The green fluorescence due to the presence of the chimera (upper left panel, marked *EGFP*) matches the staining pattern of the antibody (upper right panel, marked *Eag1.62.mAb*), as seen as yellow colour in the merged pseudo-colour image (lower panel, marked *Overlay*) Scale Bar: 20 μm. **d-g**. Light micrographs showing the immunohistochemical reaction for the monoclonal antibodies in rat hippocampus (**d**, Eag1.62.mAb; **e**, Eag1.33.mAb) and cerebellum (**f**, Eag1.62.mAb; **g**, Eag1.33.mAb), with (right column) and without (left column) pre-adsorption of the antibody to the corresponding fusion protein. The staining patterns are identical for both antibodies and are fully blocked by incubation with the corresponding epitopes, indicating that both antibodies specifically recognise the same molecular entity. Scale Bar: 50 μm.

The epitope recognised by the antibody is fully conserved between human, rat and mouse channels, and we took advantage of this situation to save valuable human material. Unfortunately, a positive signal in a western blot analysis required up to 100 μg total protein from a rat brain membrane preparation (Fig. 1a). Although no additional bands appeared in the blot and the specificity of the antibody appeared conclusive, we were unable to detect a signal on extracts from CHO cells expressing the human channel, probably due to the difficulty of obtaining large amounts of membrane proteins from cultured cells.

The selection criteria used for our antibodies make it very unlikely that another potassium channel would be recognised. All of our clones are able to discriminate between Eag1 and Eag2 (see Methods), Eag2 being the most similar potassium channel to Eag1 described so far (73% identical [19-21]). Indeed, of the nine amino acid residues that form the epitope recognised by Eag1.62.mAb, three are different in Eag2. Immunofluorescence experiments performed on cells transfected with either Eag1 or Eag2 confirmed that Eag1.62.mAb discriminates between the two (Figure 1b). We performed immunofluorescence studies on CHO cells transiently transfected with Eag1 in the pTracerCMV vector, which also expresses GFP. In these experiments, the immunohistochemical localization of Eag1 was achieved using a red-fluorescent secondary antibody (Alexa 546). Red fluorescence was restricted to cells also showing the green fluorescence of GFP, indicating that the Eag1.62.mAb antibody labelled only cells expressing Eag1 (not shown).

We then performed transient transfections with another protein chimera containing the entire Eag1 channel with the enhanced GFP fused to its N-terminus (EGFP-hEag1). The characterization of this chimera showed that the electrophysiological properties of Eag1 are preserved (data not shown). When these cells were immunostained with Eag1.62.mAb, red fluorescence co-localised with the green fluorescence of the EGFP-channel fusion protein (Figure 1c). Thus, the antibody bound only to cells that had been transfected with Eag1 and only to areas where the channel was localised. Indistinguishable staining patterns were obtained when using the monoclonal antibody against a different epitope in the C-terminus of Eag1 (Eag1.33.mAb, data not shown). Although Eag1 is an integral membrane protein (as unequivocally demonstrated by electrophysiological measurements [6,10,12,13,18,22-24,24]), we observed a strong intracellular staining that masked the cell membrane signal that was as such evident only in some cells. This result, however, is a common finding for membrane proteins and may reflect newly synthesised channels that are being transported to the membrane (e.g., [26]).

Our antibodies show cross-reactivity to rat Eag1 (99% identical to the human channel). We used this property to further characterise the specificity of the antibody, since the expression of Eag1 in rat brain has already been described [20,27,28]. We obtained staining patterns that overlapped precisely with those described for rat Eag1 in both the hippocampus (Fig. 1d,e) and cerebellum (Fig. 1f,g). Pre-incubation of the antibody with the fusion protein used to immunise the mice completely abolished staining (Figure 1d,f, right panels). Additionally, immunostaining with a different antibody (Eag1.33mAb), directed against the C-terminus of the channel, gave identical staining patterns that could also be blocked by the corresponding antigen (Figure 1e,g).

Taken together, these results strongly suggest that Eag1.62.mAb specifically recognises the Eag1 protein.

### Eag1 expression level is low in normal non-neural tissues

**Figure 2:**
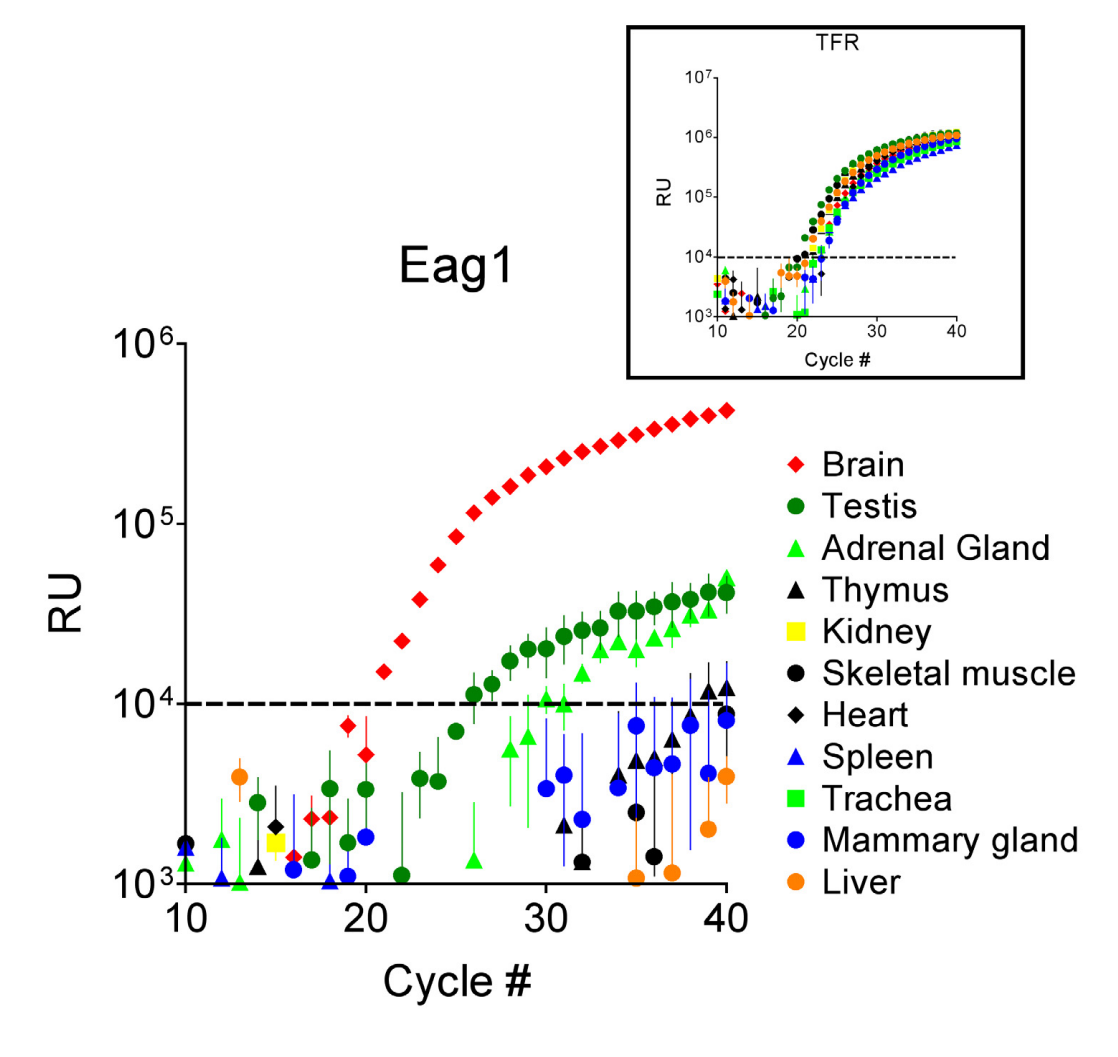
**Expression of Eag1 RNA in peripheral tissues is restricted**. Real-time PCR records on cDNA from different human RNAs. The average fluorescence obtained in three experiments (± standard error) is plotted against cycle number. The dotted line indicates the threshold values used to determine positive signals. A clear signal can be detected after 21 cycles only in brain (red diamonds), while testis (dark green circles) and adrenal glands (light green triangles) required several more cycles of amplification for the signal to reach threshold. The rest of the tissues were negative. The *Inset *shows the control amplification obtained simultaneously on the same samples using the human transferrin receptor (TFR) as a template to demonstrate RNA integrity.

In previous studies [6,11], dot blot, Northern blot and RT-PCR analyses all indicated that Eag1 is preferentially expressed in human brain. To confirm this finding and allow a more quantitative comparison, we performed real-time PCR on commercially available RNA extracted from several normal human tissues (Fig. 2). After normalising the RNA quality and tissue activity with respect to the transferrin receptor mRNA, our results confirmed the reported specific expression pattern of Eag1. The normalised levels of Eag1 expression obtained (brain = 1, see Materials and Methods) were: skeletal muscle 0.005, thymus 0.04, kidney 0.019, heart 0.003, spleen 0.0, trachea 0.046, mammary gland 0.028, adrenal gland 0.114, testis 0.124 and liver 0.0.

Total RNA from whole organs may be limited by the potential dilution of a specific mRNA from a discrete cell population. Thus, it is possible that the RNA under study may not be detectable in a particular tissue despite being highly expressed in a limited subpopulation of cells. To overcome this problem, histological techniques need to be applied.

**Figure 3:**
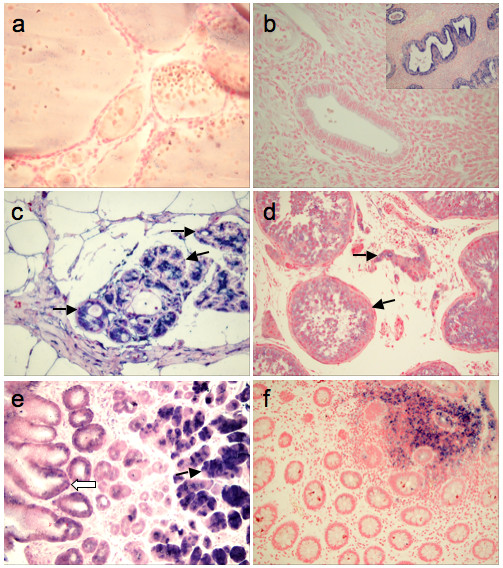
**Some normal tissues show Eag1 staining in restricted populations**. In the female reproductive system, the follicular epithelia (**a**) do not show Eag1-staining. The surface and gland epithelia of the resting endometrium are also negative (**b**). However, in proliferating and secretory activated glands, a strong Eag1-expression can be observed (**b. Inset**). In the healthy mammary gland (**c**), Eag1 signals are limited to luminal cells in the acini and ducts of the ductulo-lobular unit, while the basal cell layer is negative (**arrow**). In the testis (**d**), interstitial cells and spermatogonia within the ducti seminiferi show a weak to intermediate Eag1 expression. (**e**) The gastric corpus mucosa express little or no Eag1 (**white arrow**) except in chief cells (**black arrow**) where very strong signals are observed. In colon (**f**), the normal mucosa is negative, while mucosa-associated lymphocytes stain positive.

To determine the cellular distribution of the Eag1 potassium channel we analyzed biopsy specimens by immunohistochemistry using the Eag1.62.mAb. In good agreement with the results obtained by RT-PCR, the overall staining was absent or faint in all organs tested. A closer examination revealed that restricted cell populations with clear Eag1 reactivity do exist as described below and some examples are shown in Fig. 3. In the gastrointestinal tract, gastric gland chief cells and pancreatic acini were the only populations showing positive signals. In the male reproductive system, the spermatogenic cells were positive, in agreement with RNA expression data. In the female reproductive system, epithelial cells in both the endocervix and endometrium were moderately positive, particularly in secretory activated endometrial glands. This contrasts with the surface epithelium, which showed very low signal intensity. In non-transformed breast tissue we found variable staining of the ductular-lobular unit, in contrast to the virtual absence of Eag1 reactivity in the epithelium of the ducts. Since unaltered breast tissue is not biopsied, the tissue available was always from the vicinity of tumours or from fibrocystic proliferating changes within the gland. Bone marrow, spleen, lymph nodes, thymus and tonsil were all negative. However, we did detect positive signals in the germinal centres of lymph follicles in reactive lymph nodes. Interestingly, mast cells and tissular macrophages were found to stain positively, frequently with very strong signals. We found this property useful as a positive control for our stainings, since these cells gave us an internal estimate of the highest staining level possible for that particular preparation. Regarding the endocrine and autonomous nervous system, the anterior pituitary and the adrenal gland were stained with a low intensity both in cortex and medulla, again confirming the RT-PCR data. All cell subsets positively stained correspond to the terminal developmental stages of the respective lineages and therefore to a non-proliferating population.

### Eag1 is frequently aberrantly expressed in tumour tissues

**Figure 4:**
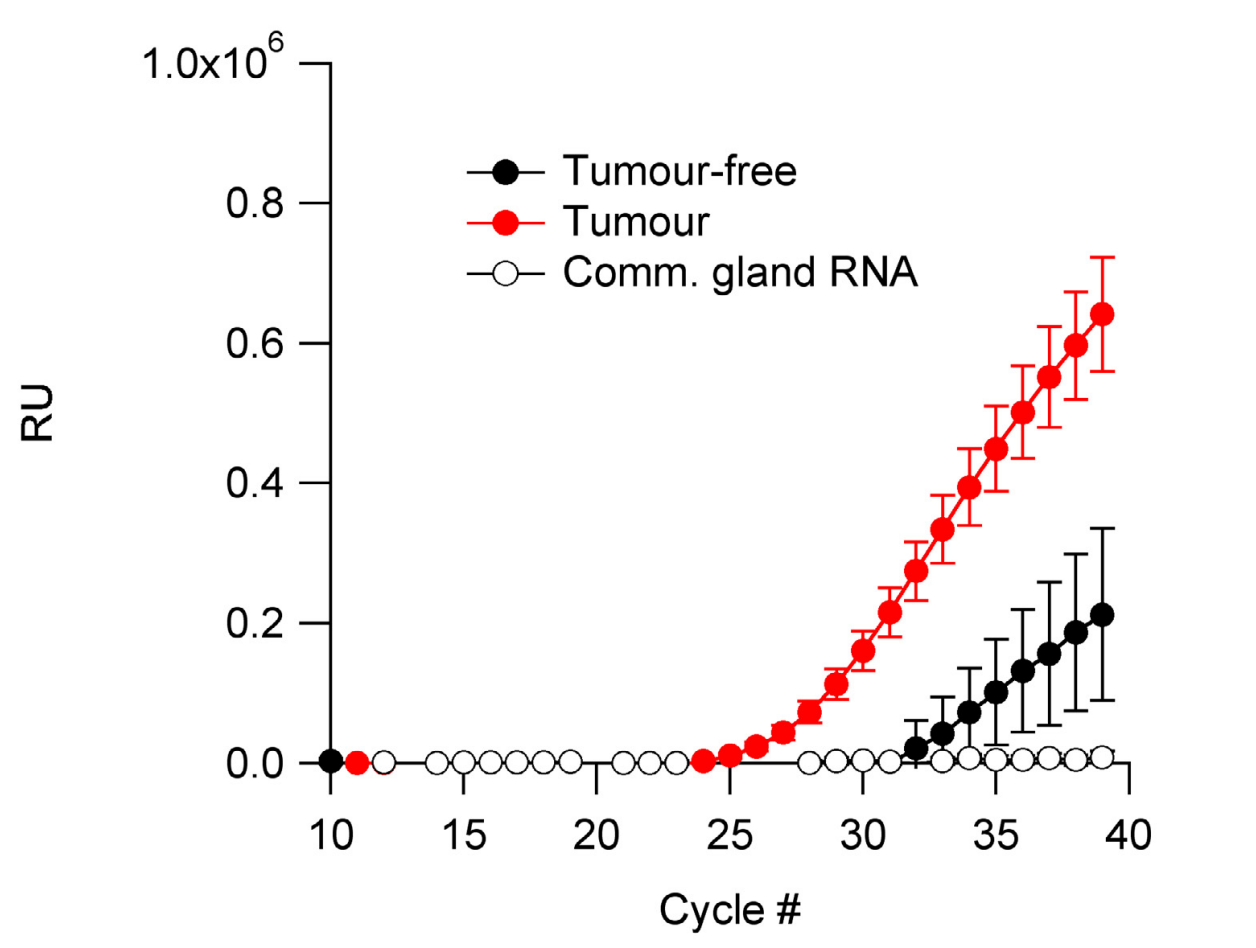
**Eag1 RNA is present in mammary tumours**. Real-time PCR amplification reveals increased Eag1 expression on cDNA obtained from human mammary gland tumours (**red trace**, N = 11), as compared with paired RNAs (**filled black circles**) from three of the cases (since only those samples were available) and commercially available normal mammary epithelium RNA (**open circles **designated comm. gland RNA). Data points represent average fluorescence units from the reporter fluorophore versus PCR cycle number. Error bars represent standard error.

We quantified the expression of Eag1 within several breast carcinomas using real-time PCR and compared it to tumour-free tissue from the same biopsy when available (Figure 4). There was a clear increase in RNA levels of Eag1 in the tumour samples as compared to the paired tumour-free tissues. The tumour-free samples also showed increased Eag1 expression over commercially available normal mammary gland RNA. This could be due either to the different sources of tissue or to elevated Eag1 expression in the vicinity of the tumour beyond the actual tumour cells.

**Figure 5:**
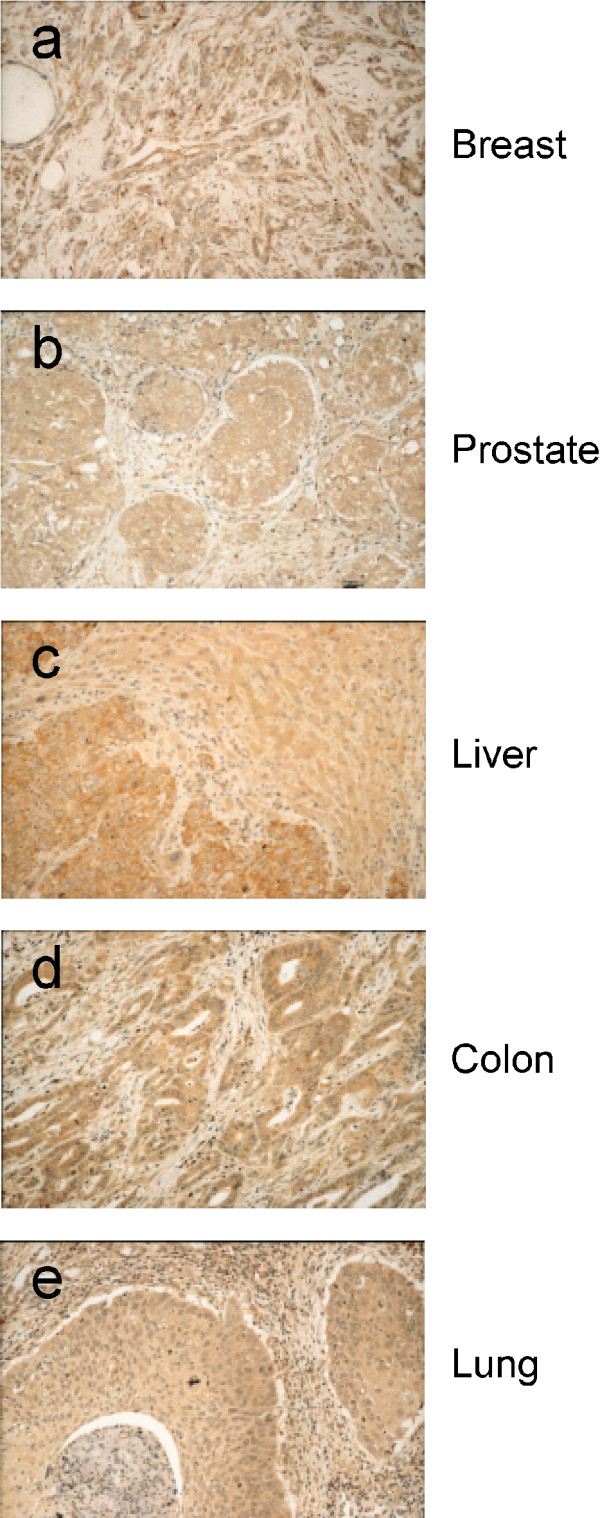
**Representative images of neoplastic tissue: Immunoperoxidase stainings using Eag1.62mAb as primary antibody**. Examples of high expression levels of Eag1 in mammary carcinoma (**a**), prostate carcinoma (**b**), hepatocellular carcinomas (**c**) colon carcinoma (**d**) or squamous cell lung carcinoma (**e**) (Hematoxylin counterstain, ×400).

**Figure 6:**
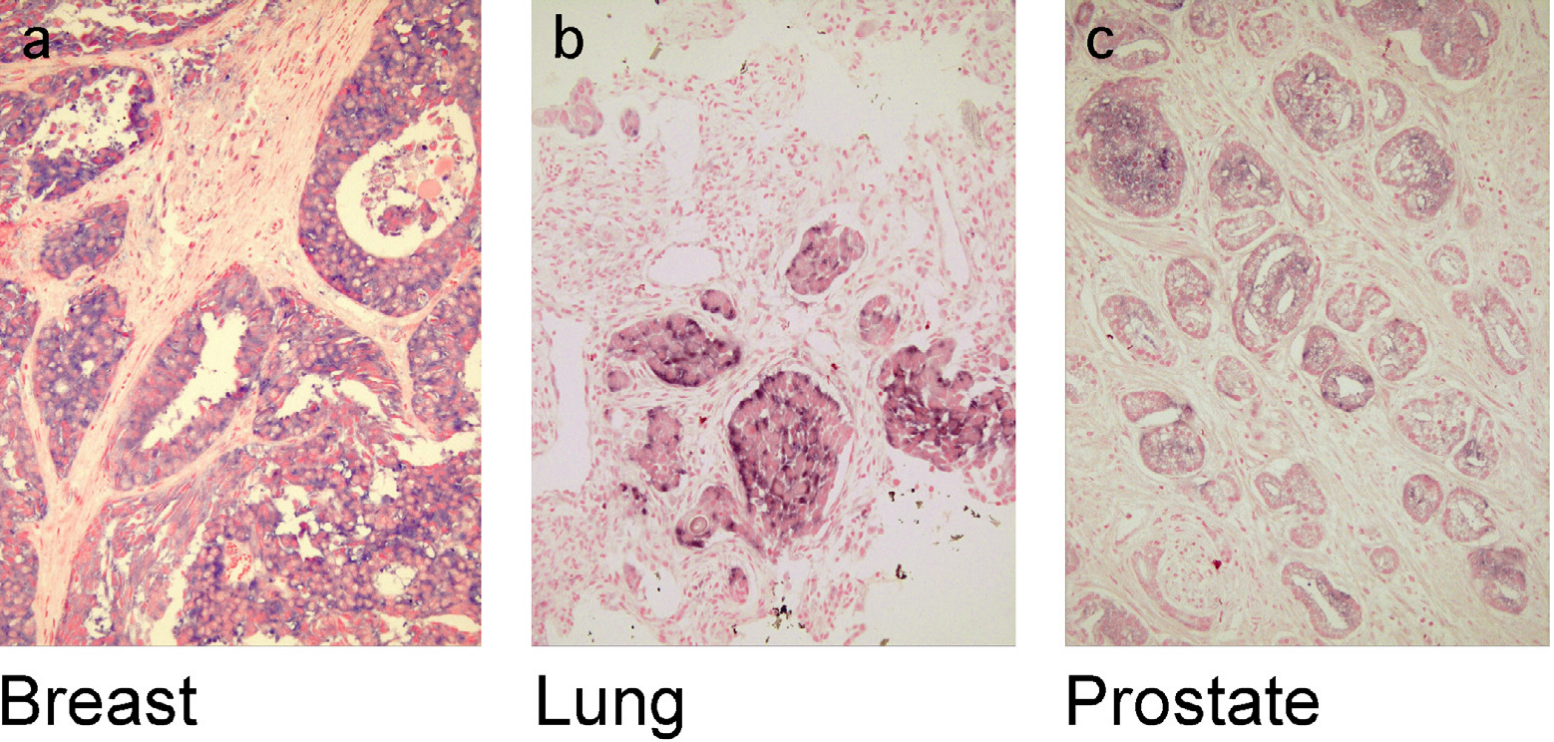
**Representative images of neoplastic tissue stained with alkaline phosphatase coupled to anti Eag1 single chain antibody**. (**a**) Ductal-invasive mammary carcinoma (**b**) Lung carcinoma (squamous cell). (**c**) Prostate carcinoma.

Immunohistochemical analysis applied to a larger number of breast carcinomas further completed and confirmed the previous RT-PCR results. Seven of eight specimens in our archive were unequivocally positive for Eag1 albeit to varying degrees. We also tested a commercial multiple tissue array containing samples from 50 breast cancer cases- all were clearly positive with Eag1 being detectable in a large proportion of the tumour cells. Additionally, we studied tumour samples from the Manitoba Breast Tumor Bank, with similar results. In contrast, the mammary epithelium was negative under both normal conditions and in the tumour-free areas in all cases. Altogether, Eag1 could be detected in 80% of the breast cancers investigated in this study (Table 1, Figures 5 and 6). We also analyzed other common human tumours with comparable results. The immunohistochemical data on tumour samples are summarised in Table 1.

**Table 1:** Immunohistochemical detection of Eag1 in tumours

**Tumour type**	**Whole antibody**		**Recombinant PhoA scFv**	
	**N**	**Positive**	**N**	**Positive**

**Oesophagus carcinoma**	8	8	12	8
**Gastric carcinoma**	10	9	14	6
**Colon carcinoma**	8	6	40	31
**Hepatocellular carcinoma**	10	10	8	5
**Gallbladder carcinoma**	5	4	1	1
**Pancreatic carcinoma**	8	6	1	1
**Renal cell carcinoma**	9	9	9	6
**Transitional cell carcinoma**	9	8	6	4
**Prostate carcinoma**	56	55	1	1
**Cervical carcinoma**	9	7	-	
**Endometrial carcinoma**	10	10	-	
**Cystadenocarcinoma of the ovary**	10	10	-	
**Breast carcinoma**	230	196	116	95
**Bronchus carcinoma**	10	9	73	41
**Thyroid papillary carcinoma**	9	9	3	2
**Basalioma, spinalioma**	10	1	-	
**Malignant melanoma**	59	22	1	1
				
**Total**	470	378 (80%)	286	202 (71%)

We next describe results of some epidemiologically relevant tumours. As previously mentioned, Eag1 is only faintly detectable in normal prostate epithelium. In contrast, 54 of the 56 analyzed cases of prostate carcinoma were strongly positive in our Eag1 immunostaining experiments. Similar results were obtained for normal bronchus and colon epithelia. Normal colon epithelium was negative or faintly positive in our experiments, while 6 of 8 colon carcinoma samples stained strongly positive (the remaining two samples were faintly positive). In the lung, we could detect some Eag1 expression in the sub mucous glands, while the bronchial epithelium was negative. In contrast, 9 out of 10 bronchus carcinomas scored highly positive (see Materials and Methods).

Liver is one of the tissues in which our methods failed to detect any Eag1 expression at the RNA level. It was also completely negative by immunohistochemistry, although under pathological conditions such as steatohepatitis we found immunohistochemical signals in hepatocytes. In contrast, 10 out of 10 liver carcinomas tested were strongly positive (Table 1, Figure 5). Together with the data from several different neoplasms, we found a very high frequency of abundant Eag1 immunoreactivity, over 90% in average. In all cases, the corresponding normal tissue from which the tumour originated was negative. Where tested, normal organs were also found to be negative for Eag1 using RT-PCR.

While performing the experiments described above, we noticed that several factors strongly influence Eag1 staining. The procedure used for tissue preservation is very critical, as it can lead to both loss of the antigen and to non-specific staining. We also noticed that the particular monoclonal antibody used loses activity with time, and can also give rise to unspecific staining. For this reason, we decided to generate a recombinant antibody with the same specificity.

Single chain antibodies can be produced from antibody molecules by joining the C-terminus of the heavy chain variable region to the N-terminus of the light chain variable region by a polypeptide spacer [29,30]. Such constructs usually have binding affinities similar to those of the native antibodies [31], and can also be produced in prokaryotic expression systems [32]. Furthermore the single chain antibody proteins can be fused to alkaline phosphatase to allow their detection by a simple and robust enzymatic colour reaction [33]. This reduces the potential for non-specific staining by eliminating the secondary antibody and the production of the single chain antibody in *E. coli *provides a more homogeneous source of material. The single chain fusion antibody was applied to brain slices and yielded staining patterns indistinguishable from those obtained with the whole antibody.

We subsequently used this recombinant antibody for the detection of Eag1 in multiple tissue arrays from different sources (see Methods), as well as tumour and normal tissues from our own collection. The results obtained using this recombinant antibody are summarised in Table 1 and Figure 6, and agree with those obtained with the conventional antibody. The normal tissues from our collection showed the same staining pattern when either of the two antibodies was used. In contrast, tumour samples from multiple tissue arrays stained with the single chain antibody produced a general decrease in the frequency of positive signals. This effect may be attributed to the lack of an amplification step when using the single chain antibody.

We detected a higher frequency of positive signals using the single chain antibody in normal tissues from multiple tissue arrays than in tissues from our archive. In contrast, the frequencies observed in tumour tissue arrays from the same commercial source were similar to the results from our archive. An explanation for this discrepancy may be the different treatment of the tissue arrays. Tumour tissue is generally obtained from biopsies and surgical specimens (and therefore undergo similar handling to the tissues from our archive), while normal tissue in the arrays frequently stems from necropsy material and therefore it is treated differently than our normal tissue (which is from biopsies and surgical origin). Therefore, suboptimal fixation can lead to false positives.

## Discussion

The emerging role of potassium channels in cancer has gained increasing interest in recent years (reviewed e.g. in [2,3,5,34,35]). Among these channels, Eag1 is unique in its restricted expression outside the CNS [6,10] while being overexpressed in tumour-derived cell lines [6,12-14,16,36] and detectable in several cases of cervical carcinoma [18] using the antibody described in this work. However, the actual frequency of expression of Eag1 in tumours remained unknown. We therefore designed and generated tools to permit the immunohistochemical detection of Eag1 in clinical specimens and used them in combination with molecular biology to address this issue.

The first problem when investigating the expression patterns of a protein is the specificity of the available tools, especially in the case of antibodies. Our selection criteria during the antibody generation process were very stringent, that is, only those clones not recognising Eag2, the closest relative to Eag1, in both ELISA and SPR experiments were pursued. The commonly accepted method used to define the specificity of an antibody is the recognition of the target protein in a western blot without evidence of any non-specific cross-reacting bands. We performed experiments using rat brain extracts and could show the absence of non-specific bands. However, similar to many other monoclonal antibodies, Eag1.62.mAb has a very low sensitivity in western blots as compared with polyclonal antibodies. We therefore used alternative approaches to confirm the selectivity of the antibody. First, immunofluorescence experiments showed that the antibody recognises human Eag1 in transfected cells but does not stain cells transfected with human Eag2. Second, the pattern of expression detected by Eag1.62.mAb in rat brain corresponds to that described in *in situ *hybridisation experiments and matches perfectly the pattern of an alternative Eag1 antibody with a completely different epitope. The staining in rat brain is completely blocked by pre-incubation of the antibody with its epitope. Taken together, these data strongly suggest the ability of Eag1 to specifically recognise its epitope while not detecting Eag2.

HERG [37] is another channel related to Eag1 showing relatively low homology to Eag1 but relevant in the context of tumour biology e.g. [38-42]. Cross-reactivity with HERG channels was therefore a concern. The absence of the specific epitope recognised by Eag1.62.mAb and the absence of a signal in smooth muscle where HERG expression is abundant [43] make it very unlikely that Eag1.62.mAb recognises HERG. In practical terms, the safest control for cross-reactivity will be achieved by testing healthy human myocardial tissue to which we has no access yet.

Many aberrantly expressed tumour-associated proteins are useful tools for the management of cancer patients [44]. The expression of such proteins has been used to make diagnostic, prognostic and therapeutic decisions. In some cases, the aberrantly expressed protein can be used for tumour vaccination (e.g., [45] for a review). However, the high frequency (>85% in 15 of 17 tumour types; Table 1) with which Eag1 was found to be overexpressed in this study of various neoplastic tissues is unusual, especially given that most molecules overproduced in neoplastic tissues [44] are ubiquitous proteins expressed also in normal tissues.

It is important to mention that although immunostaining mainly reveals cytoplasmic signals, electrophysiological measurements have detected Eag1 activity in the plasma membrane [6,10,12,13,18,22-24]. This unequivocally demonstrates surface expression of the channel. Even in transfected cells with robust current expression, it is difficult to detect membrane staining of the channel, although biotinylation experiments have shown that the channel is exposed to the external milieu [24].

It is still unclear at which stage of malignant transformation expression of Eag1 increases. Chromosomal aberrations affecting the long arm of chromosome 1 are relatively frequent. In fact of 1800 recurrent chromosome 1 aberrations, 280 directly affect region 1q32 and therefore Eag1 [46]. Since the channel itself is sufficient to induce transformation and can also increase both the growth rate and the invasiveness of experimental tumours [6], Eag1 expression may confer a growth advantage to tumour cells and permit a selective enrichment of Eag1-expressing cells. Given the elevated levels of Eag1 in various tumour tissues, it is interesting that we have previously shown that inhibition of Eag1 expression leads to a reduction of DNA synthesis in human tumour cell lines [6,15]. Whether this will also be the case in primary tumours remains to be elucidated.

We believe that our data justify further studies to qualify Eag1 as a target for clinical applications. Like Her2/Neu, Eag1 is a transmembrane protein, extracellularly accessible, involved in signal transmission and expressed in neoplastic tissues such as breast cancer, although only to a limited extent in normal tissue [47]. Her2/Neu is expressed in about 30% of breast cancers, where it has proven effective as a target for immunotherapeutic approaches [48-50]. A similar scenario is conceivable for Eag1, given its striking tumour specificity. A potential Eag1-targetted therapy would have advantages in comparison to other established therapeutic approaches. First, it could be applied to a broad spectrum of neoplasms that overexpress the Eag1 channel and thus become available to a large number of patients. Second, most normal cells expressing Eag1 are either protected by the blood brain barrier or represent terminally differentiated cells, thereby allowing more aggressive therapeutic intervention.

## Conclusion

Eag1 expression is limited outside the CNS, but is frequently expressed in tumours from diverse origin. This situation could be used in possible prognostic, diagnostic and therapeutical designs.

## Methods

### Molecular biology

Total RNA obtained from different normal tissues (Clontech Laboratories GmbH, Heidelberg, Germany) or from primary tumour biopsies (using RNeasy, Qiagen, Hilden, Germany) was reverse transcribed (SuperScript, Invitrogen, Karlsruhe, Germany) and real-time PCR was performed using the TaqMan system in an AbiPrism 7700 Sequence Detector (Applied Biosystems, Foster City, CA). The oligonucleotides used were (5'-3'):

TCTGTCCTGTTTGCCATATGATGT, CGGAGCAGCCGGACAA, and FAM-AACGTGGA-Amino C6 dT-GAGGGCATCAGCAGCCT (probe).

Transferrin receptor was used to control for RNA integrity, with the following oligonucleotides: GACTTTGGATCGGTTGGTGC, CCAAGAACCGCTTTATCCAGAT, and JOE-TGAATGGCTAGAGGGA-TAMRAdT-ACCTTTCGTCCC (probe)

A standard curve was prepared with synthetic Eag1 RNA. cRNA was prepared using standard protocols [51] from human Eag1 subcloned into the pSGEM vector (Prof. M. Hollmann, Bochum University). The exact RNA concentration after synthesis was measured by fluorescent labelling (RiboGreen, Invitrogen, Karlsruhe, Germany); different amounts of the synthetic RNA were mixed with total RNA from rat liver; cDNA was prepared from the mixture and processed by TaqMan PCR. The number of PCR cycles required to reach the detection threshold was used to determine tissue mRNA content, using the standard curve for interpolation. Relative amounts of RNA were obtained with respect to the brain Eag1 mRNA content which was set to 1.0. The amplified transferrin receptor was used to normalise the quantity of mRNA from the tissue sample. The constructs used for transient transfection were the already described pTracerCMVhEAG1[6] and chimera EGFP-hEag1, constructed by introducing an EcoRV site at position 1 of the Eag1 open reading frame (QuickChange Site Directed Mutagenesis Kit, Stratagene, Amsterdam, The Netherlands) and subcloning into the pEGFPC2 vector (Invitrogen, Karlsruhe, Germany).

### Antibody design and characterization

A fusion protein containing an area close to the putative pore region (residues 374 to 452, Eag1.62.mAb) and another in the C-terminus of Eag1 (residues 872 to 932, Eag1.33.mAb) was used for immunization. The fusion protein was cloned in the pET32 vector (Novagen, Madison WI, USA), which contains a thioredoxin tag to enhance solubility and a poly-histidine tail that was used for purification after overexpression in *E. coli*.

Hybridomas were generated by standard techniques (mice immunization, fusion, selection of positive clones and two cloning steps; BioGenes GmbH, Berlin, Germany). The epitope recognised by each supernatant and the ability to differentiate between Eag1 and the most closely related protein known (Eag2 [19-21]) were determined by surface plasmon resonance [52]. Those supernatants with the best performance in this test were then used to examine human brain tissues immunohistochemically and the hybridomas giving the expected staining patterns were subcloned. The antibodies used in this study were subsequently purified by affinity chromatography, first on a protein A column and then on a second affinity column with the fusion protein used for immunization. The activity of the antibody was again checked by surface plasmon resonance prior to use.

To generate a single chain, alkaline phosphatase (PhoA) fused antibody, the antibody cDNA cloning was achieved by first determining the subtype of the murine antibodies (IgG κ2b) using IsoStrip (Roche Applied Science, Mannheim, Germany). Total RNA was prepared from the hybridoma cells (RNeasy kit, Qiagen, Hilden, Germany) and translated into cDNA (SMART PCR cDNA Synthesis Kit, Clontech, Heidelberg, Germany). Subsequently the genes of the light and heavy chains expressed by the hybridomas were amplified by PCR using Pwo polymerase (Roche, Mannheim, Germany) with the following primers:

GTAACAACGCAGAGTACGCGGG and either TCATTTACCCGGAGACCGG (heavy chain) or CTAACACTCATTCCTGTTGAAGCTC (light chain).

The PCR products were subcloned into pBKS+ and sequenced. The variable regions of both chains were identified by sequence comparison [53] and fused by PCR with a linker sequence between the 3'-end of the heavy chain variable region and the 5'-end of the light chain variable region (Gly-Gly-Gly-Gly-Ser).

Primer sequences were:

TCTGGAGGTGGAGGTAGTGGGGGAGGAGGTTCAGATGTTGTGATGACCCAAACTCC and CCCACTACCTCCACCTCCAGAGCCTCCCCCTCCTGAGGAGACGGTGACTGAGG (heavy chain) and

TCTGGAGGTGGAGGTAGTGGGGGAGGAGGTTCAGATGTTGTGATGACCCAAACTCC and GGCCTAATCGGCCCGTTTGATTTCCAGCTTGGTG (light chain). The fusion was then performed using the first and the last of these sequences.

To produce the PhoA fusion protein, the single chain antibody fragment was amplified and a 5' SalI and a 3' NdeI restriction site introduced. This fragment was cloned (blunt end) into an EcoRV-digested pBKS+ vector prior to subcloning (SalI/NdeI) into pQUANTagen(kx) which contains the alkaline phosphatase sequence. Finally, the single chain antibody fragment – PhoA fusion protein was subcloned into the expression vector pASK-iba2 (using SmaI-SalI sites introduced by PCR)

The fusion protein was obtained from inclusion bodies produced in E. coli (triggered by treatment of anhydro-tetracycline 200 ng/ml). The cells were harvested in 100 mM Tris/HCl, 100 mM NaCl, pH 8.0, 8 M urea and sonicated at 0° until lysis was complete and the solution appeared clear. The lysate was stirred overnight at 4°C and then dialyzed (cut-off 30 kDa) against 6, 4, 2, 1 and 0 M urea in extraction buffer. The solution was then centrifuged at 18000 ×g and stored at 4°C with 0.02 % (w/v) sodium azide. The preparation was characterised by SDS-PAGE, PhoA activity and ELISA to determine the relative activity of the fusion protein compared to the whole murine antibody.

### Immunohistochemical methods

Transfected cells growing on glass coverslips were fixed (4% p-formaldehyde in PBS, 5 min at 4°C), permeabilised with Triton X100 (0.5%, 10 min), blocked (10% BSA, 30 min) and incubated for 2 h with Eag1.62.mAb (1 μg/ml) or Eag1.33.mAb (1 μg/ml). The secondary antibody (AlexaFluor 546 Goat anti-Mouse IgG, Invitrogen, Karlsruhe, Germany) was used at a 1:1000 dilution (30 min) and the preparations were examined by confocal microscopy.

Two postnatal-day 21 Sprague Dawley rats were anesthetised with a mixture of ketamine HCl (Ketaset; 100 mg/ml; Fort Dodge Laboratories, Inc., U.S.A.) and xylazine (Rompun; 20 mg/ml; Mile, Inc., U.S.A.) at 0.1 ml/100 g body weight. The animals were transcardially perfused with 4% p-formaldehyde in 0.12 M phosphate buffer (pH 7.2). After perfusion, the brains were removed, fixed for an additional hour at 4°C, rinsed three times with PBS and stored overnight at 4°C. Coronal and sagittal sections (400–500 μm) were cut in cold PBS using a vibratome (Leica, Vienna, Austria). Slices were incubated for 1 h with 10% normal goat serum in PBS, then with primary hEag1 antibody (Eag1.62.mAb: 2 μg/ml; Eag1.33.mAb: 4 μg/ml) in PBS overnight at 4°C and processed using the avidin biotin-peroxidase system (Vectastain kit, Vector Laboratories, Burlingame, CA). Antibody binding was visualised using 3'-3-diaminobenzidine tetrahydrochloride (DAB; DAB substrate kit for peroxidase, Vector Laboratories). Controls either omitted the primary antibody or incubated the primary antibody with the corresponding fusion protein (10 μg/ml final concentration) at 4°C for 24 h prior to following the procedure as described above. Sections were analysed with a Zeiss Axiophot microscope.

For the immunohistochemical detection of Eag1 potassium channels in human tissues, formalin-fixed and paraffin-embedded biopsy specimens from our own archive, samples from the Manitoba Breast Tumor Bank [54] and multiple tissue arrays (BioCat, Heidelberg, Germany) were used. Optimal staining conditions and antibody dilutions were determined using formalin-fixed and paraffin-embedded tissue samples from human cerebral cortex. Antigen retrieval was performed in a microwave oven in 10 mM citrate buffer (pH 6.0) at 700 W for at least 15 min. Slides were incubated overnight in a humidified chamber at 4°C with Eag1.62.mAb followed by incubation with the Envision Peroxidase System and DAB (DAKO, Hamburg, Germany). Two operators independently evaluated antigen expression, denoting it either as "negative" (0), "positive" (1) or "strongly positive" (2) as appropriate.

For immunodetection of the single chain antibody fragment alkaline phosphatase fusion proteins, the previous protocol was modified to use the BCIP/NBT (Roche, Mannheim, Germany) substrate and the sample was counterstained with nuclear fast red (DAKO, Hamburg, Germany). Two slides of different multiple tissue arrays were evaluated independently by two researchers using the scoring system described above.

Processing of consecutive sections from the same block in different days resulted in identical staining, illustrating the reproducibility of the system.

## Competing interests

LAP and WS are shareholders of a company interested in the exploitation of ion channels as anticancer targets.

## Authors' contributions

All authors participated in the experimental design and interpretation, BH, RMW, FMQ, HK, AS, MER, SM, TS, MJ and LAP performed experiments, and BH, HJR, WS and LAP wrote the manuscript.
